# Editorial: Ecology, evolution, and biodiversity of microbiomes and viromes from extreme environments

**DOI:** 10.3389/frmbi.2025.1604002

**Published:** 2025-04-24

**Authors:** Gareth Trubl, Lucie Malard, Janina Rahlff

**Affiliations:** ^1^ Physical and Life Sciences Directorate, Lawrence Livermore National Laboratory, Livermore, CA, United States; ^2^ Department F.-A. Forel for Environmental and Aquatic Sciences, Faculty of Sciences, University of Geneva, Geneva, Switzerland; ^3^ Centre for Ecology and Evolution in Microbial Model Systems (EEMiS), Department of Biology and Environmental Science, Linnaeus University, Kalmar, Sweden; ^4^ Aero-Aquatic Virus Research Group, Faculty of Mathematics and Computer Science, Friedrich Schiller University Jena, Jena, Germany; ^5^ Leibniz Institute on Aging - Fritz Lipmann Institute (FLI), Jena, Germany

**Keywords:** microbial communities, extreme environments, metagenomics, viromics, adaptation, biotechnology

Ecology, evolution, and biodiversity of microbiomes and viromes in extreme environments are key areas of research that explore how microbial communities adapt, survive, and thrive under harsh conditions. The studies published in our Research Topic advance our understanding of microbial and viral diversity, evolutionary processes, and the ecological roles of these communities, with implications for biotechnology, climate resilience, and even astrobiology.

Microbial life in extreme environments is shaped by the interplay between environmental pressures and adaptive strategies. Deep-sea hydrothermal vents serve as natural laboratories for studying microbial evolution and adaptation, offering analogs for primordial biochemical processes. A study by Alian et al. on the Lost City Hydrothermal Field near the Mid-Atlantic Ridge investigated microbial communities thriving within its actively venting carbonate chimneys. Their findings revealed that bacterial communities—including *Desulfotomaculum, Sulfurovum, Thiomicrorhabdus*, and *Serpentinicella*—exhibit high levels of microdiversity, driven by mineral composition rather than just vent fluid chemistry. Shotgun metagenomic analyses further showed that genes involved in carbon, methane, nitrogen, and sulfur cycling are closely linked to specific mineralogical characteristics, highlighting the importance of microbe-mineral interactions in shaping microbial community structure and function.

Moving from the ocean depths to the terrestrial extreme of hypersaline environments, Ionescu et al. explored microbial biofilms in Dead Sea underwater springs, where bacteria must rapidly adjust to unpredictable fluctuations in salinity, pH, and oxygen. Metagenomic analysis of an enrichment culture identified key taxa—*Prosthecochloris, Flexistipes, Izemoplasma, Halomonas*, and *Halanaerobium*—that employ both the “salt-in” and “salt-out” osmoregulation strategies, allowing scalable adaptation similar to halophilic archaea. Genomic data indicate that while *Izemoplasma* relies on compatible solutes, others use mechanosensitive channels for rapid ionic adjustments. The findings challenge the conventional dichotomy of osmoregulation strategies and suggest a flexible mixed approach, expanding our understanding of microbial resilience in fluctuating environments.

Furthermore, the study by Tammert et al. explored the impact of increasing salinity on freshwater sediment and water bacterial communities, an increasing concern from industrial salt discharges and climate change-induced salinization. They showed that bacterial communities, especially from sediments, are highly resistant to increasing salinity but that the community adapts when faced with long-term exposure. For instance, the abundance of *Hydrogenophaga* increased as salinity increased. This shift indicates that although the community withstands salinity changes, individual species may be differentially affected.

Beyond extreme salinity, extreme hydrocarbon environments also harbor diverse microbial life with unique metabolic adaptations. Beilig et al. advanced our understanding of anaerobic oil degradation by employing reverse stable isotope labeling to quantify microbial mineralization rates in light oil reservoirs. Oil reservoirs contain microbial communities capable of degrading crude oil over geologic timescales, yet the actual rates of degradation remain poorly constrained. By incubating formation water from a newly drilled well, they estimated a degradation rate of 15.2 mmol CO_2_/mol CH_2_ per year—closely aligning with modeled reservoir degradation predictions. Their study highlights the role of sulfate-reducing and fermentative microbial communities, with Desulfobacterota, Thermotogota, and Bacteroidota emerging as key taxa, while also demonstrating the potential of reverse stable isotope labeling as a powerful tool to measure microbial degradation in complex hydrocarbon environments.

Moving into the cold, nutrient-poor conditions of icy landscapes, Faber et al. investigated the meltwater microbial communities on a glacial ice surface in Alaska. The metagenomic analysis revealed unique communities enriched in Gammaproteobacteria, Dothideomycetes, Microbotryomyetes and algae, with three times more fungal genes, associated homeostasis, cellular regulation, and stress responses. These findings contribute to understanding microbial life in glacial ecosystems and provide insights into their adaptation to extreme, cold environments.

Continuing our journey in the cold Swiss Alps, Busi et al. examined the stability of benthic biofilms in proglacial streams, focusing on interactions between bacterial and fungal communities. The authors showed that co-occurrence networks were relatively stable and that the identified bacterial and eukaryotic keystone taxa were critical for network stability. Interestingly, low-abundance taxa played significant roles in stabilizing microbial communities. These findings suggest that microbial functions of keystone taxa may be essential to community stability.

Still in the Swiss Alps, Peter et al. investigated the viral communities in sediment-derived benthic biofilm communities from proglacial streams, revealing that viral community composition closely mirrors bacterial diversity, with high host specificity and potential functional roles in microdiverse bacterial clades through auxiliary metabolic genes (AMGs), such as those involved in the metabolism of cofactors and vitamins. The work demonstrates bacteria-phage interactions and ecological dynamics in streams influenced by glacial meltwaters, which are considered extreme environments due to their harsh conditions, such as near-freezing temperatures and low nutrient availability (ultra-oligotrophy). They highlight that viruses also play a critical role in extreme ecosystems by shaping microbial diversity, influencing host metabolism, and driving biogeochemical cycles.

Finally, Oliveira et al. conducted research on the Great Amazon Reef System (GARS), revealing how sponges in this unique, low-light, sediment-rich environment rely on their microbiomes to support biogeochemical cycles and host nutrition. The analysis of metagenome-assembled genomes (MAGs) from GARS sponges, particularly the candidate phylum Latescibacterota, uncovered metabolic pathways related to nutrient cycling, pollutant degradation, and bioactive compound production. These findings emphasize the role of microbial symbionts in supporting host survival in extreme environments and highlight their promising potential for bioremediation and biotechnological applications.

Together, these studies provide a comprehensive look at how microbial communities and their viral counterparts adapt to extreme conditions, from the deep sea and hypersaline springs to hydrocarbon reservoirs, glacial meltwaters, and mesophotic reefs. Despite the diversity of these environments, common themes emerge: microbial adaptation is driven by selective pressures that shape metabolic flexibility, symbiotic relationships, and evolutionary innovation. Whether through microbe-mineral interactions in hydrothermal vents, scalable osmoregulation in fluctuating salinities, or virus-host dynamics in nutrient-limited streams, these studies collectively advance our understanding of life’s resilience in extreme habitats. By integrating genomic, geochemical, and isotopic approaches, this Research Topic provides novel insights into microbial and viral evolution, with broader implications for Earth’s biogeochemical cycles, ecosystem stability, and even the search for life beyond our planet.

